# Mycobacterium tuberculosis co-infection is associated with increased surrogate marker of the HIV reservoir

**DOI:** 10.1186/s12981-020-00320-0

**Published:** 2020-10-19

**Authors:** Jingna Xun, Tangkai Qi, Lei Zou, Qi Tang, Yinzhong Shen, Junyang Yang, Luman Xie, Yongjia Ji, Renfang Zhang, Li Liu, Jiangrong Wang, Corky Steinhart, Zhenyan Wang, Yang Tang, Wei Song, Jianjun Sun, Juan Cheng, Xiaoqin Le, Huanmei Wu, Xiaoqing He, Rong Chen, Jun Chen, Hongzhou Lu

**Affiliations:** 1 Scientific Research Center, Shanghai Public Health Clinical Center, Fudan University, Shanghai, China; 2 Department of Infection and Immunity, Shanghai Public Health Clinical Center, Fudan University, 2901 Caolang Road, Shanghai, 201508 China; 3 Department of Infectious Disease, Yancheng Second People’s Hospital, Jiangsu, China; 4 Infectious Disease Clinic, Longtan Hospital of Guangxi Zhuang Autonomous Region, Guangxi, China; 5 CAN Community Health, Florida, USA; 6 The University of Central Florida College of Medicine, Florida, USA; 7 Department of Infectious Disease, HuaShan Hospital Affiliated to Fudan University, Shanghai, China

**Keywords:** HIV, Tuberculosis, Reservoir, Interleukin-7, Indoleamine 2, 3-dioxygenas

## Abstract

**Background:**

Tuberculosis (Tb) is the most frequent opportunistic infection among people living with HIV infection. The impact of Tb co-infection in the establishment and maintenance of the HIV reservoir is unclear.

**Method:**

We enrolled 13 HIV-infected patients with microbiologically confirmed Tb and 10 matched mono-HIV infected controls. Total HIV DNA in peripheral blood mononuclear cells (PBMCs), plasma interleukin-7 (IL-7) concentrations and the activities of indoleamine 2,3-dioxygenase (IDO) were measured for all the participants prior to therapy and after antiretroviral therapy (ART).

**Results:**

After a duration of 16 (12, 22) months’ ART, patients co-infected with Tb who were cured of Tb maintained higher levels of HIV DNA compared with mono-HIV infected patients [2.89 (2.65- 3.05) log_10_ copies/10^6^ cells vs. 2.30 (2.11–2.84) log_10_ copies/10^6^ cells, *P *= 0.008]. The levels of on-ART HIV DNA were positively correlated with the baseline viral load (*r *= 0.64, *P *= 0.02) in Tb co-infected group. However, neither plasma IL-7 concentration nor plasma IDO activity was correlated with the level of on-ART HIV DNA.

**Conclusions:**

Tb co-infection was associated with the increased surrogate marker of the HIV reservoir, while its mechanism warrants further examination.

## Background

Currently, there are 36.9 million people living with HIV (PWH), including approximately 1.8 million new cases of infection each year [1]. Antiretroviral therapy (ART) substantially decreases the mortality of HIV-infected patients and improves the quality of life of PWH [2, 3]. The main barrier in eradicating HIV infection is the presence of the HIV reservoir [4], which refers to the persistence of replication-competent provirus among PWH undergoing long-term effective ART [5]. The mainly site for the HIV reservoir is normally referred to CD4 + T cells, dendritic cells (DCs), and macrophages [6]. Chomont et al. estimated that the half-life of HIV latent cells was 39.5 months, and it was speculated that 65.7 years of effective ART would be needed to remove 1 million cells with latent HIV infection [7]. There were two different hypotheses about the viral naïve persistence mechanism of HIV reservoir, one is the antigen-induced proliferation and the other is homeostatic proliferation of latent cells mediated by cytokine [7, 8].

People living with HIV are 15–22 times more likely to develop TB than persons without. TB is the most common presenting illness among PWH, including among those taking antiretroviral treatment, and it is the major cause of HIV-related deaths. In 2018, approximately 862,000 PWH estimated to have fallen ill with Tb [9]. Given that Tb is one of the most common complications of HIV infection, its potential impact on the HIV reservoir should be evaluated. However, studies on the impact of Tb co-infection on HIV reservoir are limited, preventing HIV/Tb co-infected patients benefiting from the eradication of HIV [4].

Tb is known to promote HIV replication and may accelerate HIV progression [10–12]. Tb is associated with higher HIV viral load in blood and cerebrospinal fluid as well as increased genetic heterogeneity of virus quasispecies compared to HIV mono-HIV infected patients [13]. Meanwhile, Tb co-infection is also associated with increased levels of plasma IL-7 [14], which is essential in the establishment and maintenance of the HIV-1 reservoir [15, 16]. In addition, Tb is also characterized by higher immune activation as evidenced by elevated levels of inflammatory markers including the activity of indoleamine 2,3-dioxygenase (IDO), which has been associated with the size of the HIV reservoir [17, 18]. Therefore, it is reasonable to hypothesize that Tb co-infection could increase the size of the HIV reservoir by the cytokine- mediated homeostatic proliferation pathway. To this end, we quantified the total HIV DNA, the level of plasma IL-7 concentration and the plasma IDO activities among PWH with or without Tb to determinate the impact of Tb co-infection on the size of the HIV reservoir.

## Materials and methods

### Patients and samples

Ten mono-HIV-infected patients and thirteen HIV/Tb co-infected patients who were living with HIV ART-naïve treated at the Shanghai Public Health Clinical Center (SPHCC) were enrolled in this study. Microbiological examination included modified acid-fast smear and solid culture on the modified Roche medium for sputum, secretions and feces, as well as liquid culture with BD-BACTEC-9120 for blood samples. ELISA was used to detect MPB64 protein in any positive cultures, which is specific for MTB. Tb was diagnosed based on the following: (a) a patient had a positive culture and MPB64 protein or (b) a patient was smear positive and culture-negative for Mycobacterium. Mono-HIV infected patients were selected from a cohort of asymptomatic PWH after matching for sex, age, CD4 T cell count, and the CD4/CD8 ratio, all the patients received initiated first-line ART for a median duration of 16 (12, 22) months. PWH with Tb, hepatitis B virus (HBV) infection, hepatitis C virus (HCV) infection, syphilis or any history of other opportunistic infections/cancers were excluded. HIV RNA for the two groups was not matched as Tb is known to be associated with higher HIV viral loads [13].

Standard anti-Tb treatment was immediately initiated after the diagnosis was made and lasted for more than 6 months, while ART was begun after 2 or 8 weeks of anti-Tb treatment initiation among HIV/Tb co-infected patients. The ART regimen consisted of two nucleotides reverse transcriptase inhibitors (NRTIs) combined with either efavirenz or lopinavir/ritonavir. Peripheral blood samples were collected by using EDTA Routine blood tube at baseline and more than 1 year’s ART. Plasma and peripheral blood mononuclear cells (PBMCs) were isolated as per the manufacturers instruction and stored at −80 °C.

### HIV DNA measurement

Genomic DNA was extracted from PBMCs utilizing the Qiagen DNA Blood Mini Kit (QIAGEN, Valencia, CA), and HIV DNA levels were determined as previously described [18]. Briefly, double fluorescence-based, real-time quantification polymerase chain reaction (PCR) with the HIV-1 DNA primer probes and the human cell actin gene region primer probes were used. Both HIV DNA and cells were amplified concurrently during the mono-tube PCR amplification reaction. The standard curve of quantitative HIV DNA and cells was obtained simultaneously according to the quantitative reference. Peripheral blood samples were collected by using EDTA Routine blood tube at baseline and more than 1 year’s ART. HIV DNA copy numbers were subsequently divided by the cell number and the product was multiplied by 10^6^ to obtain the HIV DNA content (unit: copies/10^6^ cells) in per 10^6^ cells.

### Quantitation of plasma IL-7 concentrations

Plasma IL-7 concentrations were measured using the Human Interleukin-7 (IL-7) Quantikine HS ELISA Kit (R&D Systems, Minnesota, USA) and recorded using the BIO-RAD iMark Microplate reader. Concentrations were calculated from the respective standard curves by applying 4-parametric logistic regression. The assay range of plasma in this kit was 0.3–16 pg/ml.

### Quantification of plasma kynurenine and tryptophan levels as a measure of IDO activity

Plasma kynurenine and tryptophan levels were quantified using the Human Kynurenine ELISA Kit (MyBioSource, San Diego, USA) and the Human Tryptophan ELISA Kit (MyBioSource, San Diego, USA). IDO activity was calculated as the plasma kynurenine/tryptophan ratio (K/T ratio) [18, 19].

### Statistical analysis

Clinical data was presented as means with standard deviation or medians with interquartile ranges according to the normal distribution of data performed by the Shapiro–Wilk test. Students’*t* test and standard non-parametric tests were used to compare the differences between the two groups. Levels of total HIV DNA below the lower limit of detection were deemed as 10 copies/10^6^ PBMCs. Pearson or Spearman rank tests were used to measure the correlations between on-ART HIV DNA and other variables according to the distribution of the data. All the data were analyzed with SPSS version 23.0 (IBM Corporation, Armonk, USA), and *P* value less than 0.05 was considered statistically significant for all tests.

## Results

### Demographics and clinical characteristics of the study participants

Most of the participants were male (73.91%, 17/23), with a mean age of 41.78 ± 11.10 years. Most of them were at the late stage (CD4 T cell count < 200 cells/ul) [20] of diseases [CD4 T cell count: 118.00 (48.00–193.00) cells/ul; CD4/CD8 ratio: 0.25 (0.08–0.37)]. HIV/Tb co-infected patients had higher viral loads than mono-HIV infected patients [5.77 (5.23–6.00) log_10_ copies/ml vs. 4.57 (4.36–5.35) log_10_ copies/ml, *P *= 0.003] (Table 1).

**Table 1:** Demographic and clinical characteristics of the study population

Characteristics	Mono-HIV infected group (n = 10)	HIV/Tb co-infected group (n = 13)	*P*-value
Age (years), mean ± SD	39.50 ± 11.28	38.00 ± 11.09	0.40
Male	80.00%	69.23%	0.66
Baseline CD4 T cell count (cells/μl), median (IQR)	152.00 (44.00–252.00)	101.00 (55.00–173.00)	0.41
Baseline CD4/CD8 T cell ratio	0.20 (0.07–0.48)	0.26 (0.08–0.36)	0.99
EFV based	70.00%	92.31%	0.24
Duration of ART (Months)	12.15 (11.53–14.91)	18.00 (17.00–23.00)	*<0.0001*
On-ART CD4 T cell count (cells/μl), median (IQR)	453.00 (304.75–475.75)	283.00 (215.00–388.50)	*0.04*
On-ART CD4/CD8 T cell ratio	0.54 (0.36–0.88)	0.30 (0.16–0.45)	*0.03*
Baseline viral load (Log10 copies/ml), median (IQR)	4.57 (4.36–5.35)	5.77 (5.23–6.00)	*0.003*

Italic values (*P* < 0.05) indicate significant difference between the two groups

SD: standard deviation; IQR: Interquartile Range

### Tb co-infection is associated with higher levels of On-ART HIV DNA

**Fig. 1:**
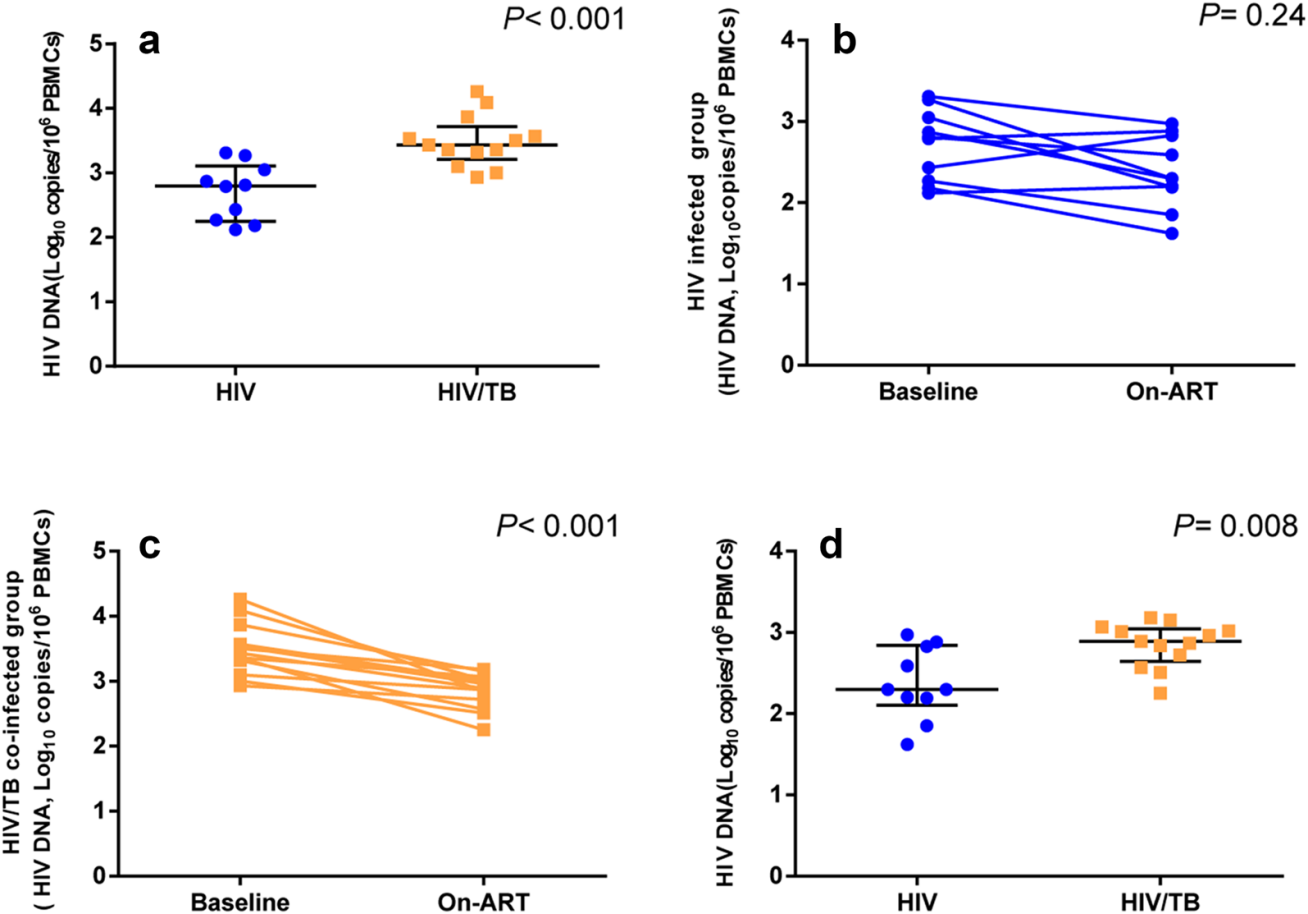
The levels of HIV DNA differ between mono-HIV infected patients and HIV/Tb co-infected patients. **a** The levels of HIV DNA in the mono-HIV infected group and HIV/Tb co-infected group at baseline. **b, c** Change in the level of HIV DNA in each group after more than 1year’s ART. **d** The levels of HIV DNA in the mono-HIV infected group and HIV/Tb co-infected group after ART. All the data analyzed by non-parametric test

At baseline, the levels of HIV DNA were higher in HIV/Tb co-infected patients than mono-HIV infected patients [3.43 (3.21–3.72) log_10_ copies/10^6^ cell VS. 2.80 (2.25–3.11) log_10_ copies/10^6^ cells, P < 0.001, Fig. 1a]. After a median 16 (12, 22) months of ART, viral loads were below the limit of detection (< 20 copies/ml) in all the patients. Tb was also cured in all the HIV/Tb patients after a standard 6-month anti-Tb therapy. The CD4 T cell count and the CD4/CD8 ratio in HIV/Tb co-infected group were lower than in mono-HIV infected group following ART [CD4 T cell count: 283.00 (215.00–388.50) cells/μl vs. 453.00 (304.75–375.75) cells/μl, *P *= 0.04; CD4/CD8 ratio: 0.30 (0.16–0.45) vs. 0.54 (0.36–0.88), *P *= 0.03] (Table 1). The levels of HIV DNA in the mono-HIV infected group have not significant difference between baseline and on-ART (average decline rate: 11.66%, *P *= 0.24, Fig. 1b), but significantly decreased in HIV/Tb co-infected patients after ART (average decline rate: 17.69%, *P *< 0.001; Fig. 1c). However, HIV DNA was still higher in the HIV/Tb co-infected group as compared to that in mono-HIV infected group [2.89 (2.65–3.05) log_10_ copies/10^6^ cells vs. 2.30 (2.11–2.84) log_10_ copies/10^6^ cells, *P *= 0.008, Fig. 1d].

### Factors associated with the levels of HIV DNA on ART

**Fig. 2:**
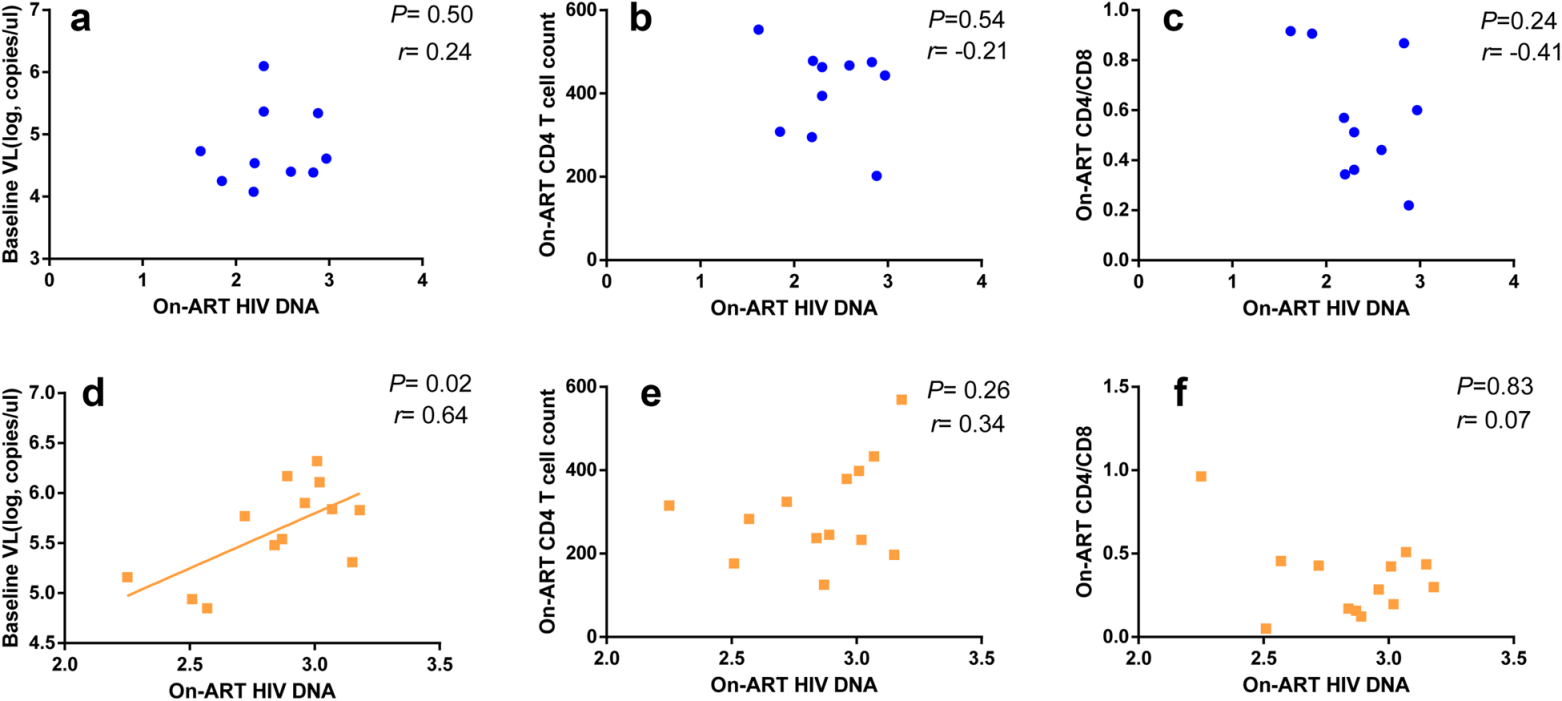
Correlations between the levels of on-ART HIV DNA and factors (baseline viral load, on-ART CD4 cell count and on-ART CD4/CD8 ratio). The correlation analyzed between the levels of on-ART HIV DNA and baseline viral load (**a**), on-ART CD4 cell count (**b**), and on-ART CD4/CD8 ratio (**c**) in mono-HIV infected group. Similarly, the correlation analyzed between the levels of on-ART HIV DNA and baseline viral load (**d**), on-ART CD4 cell count (**e**), and on-ART CD4/CD8 ratio (**f**) in HIV/Tb co-infected group. All the data analyzed by non-parametric test. The blue dot represents mono-HIV infected patients and the orange dot represents HIV/Tb co-infected patients

There was no significant correlation between baseline viral loads, on-ART CD4 T cell count, on-ART CD4/CD8 ratio and the levels of on-ART HIV DNA in the mono-HIV infected group (Fig. 2a–c). However, in the HIV/Tb co-infected group, the baseline viral loads were positively correlated with on-ART HIV DNA (*r *= 0.64, *P *= 0.02, Fig. 2d), total HIV DNA in PBMCs.

### Correlation of plasma IL-7 concentrations with the levels of on-ART HIV DNA

**Fig. 3:**
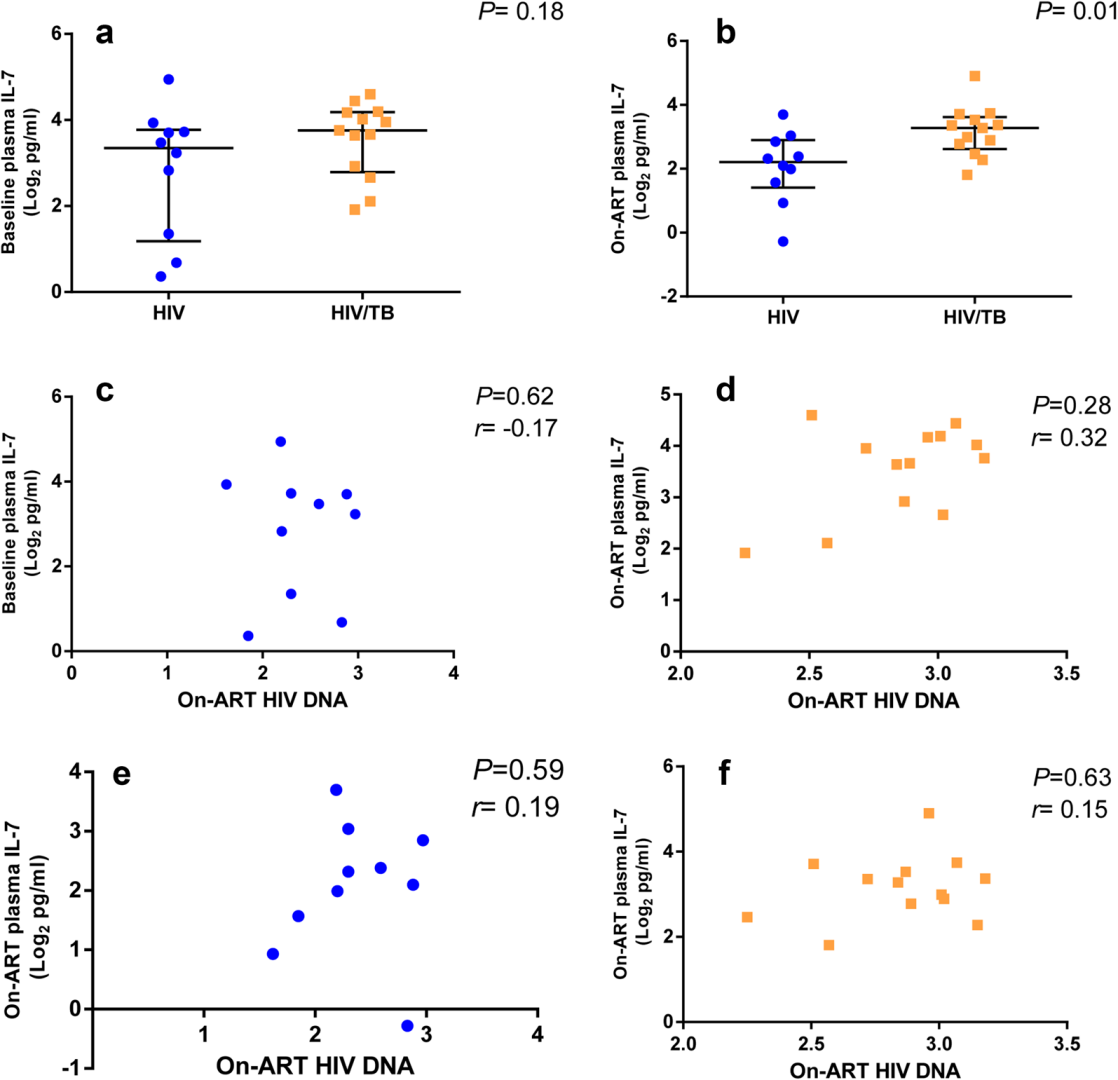
Correlations of plasma IL-7 concentrations with the levels of on-ART HIV DNA. The plasma IL-7 concentrations in the two groups group at baseline (**a**), and after ART (**b**). The correlations between the levels of on-ART HIV DNA and the plasma IL-7 concentrations both at baseline and after ART in the mono-HIV infected group and HIV/Tb co-infected group, respectively (**c−f**). All the data analyzed by non-parametric test. The blue dot represents mono-HIV infected patients and the orange dot represents HIV/Tb co-infected patients

There were no differences of plasma IL-7 concentrations in the two groups at baseline [mono-HIV infected group: 3.35 (1.18–3.77) log_2_ pg/ml, HIV/Tb co-infected group: 3.76 (2.79–4.18) log_2_ pg/ml, *P *= 0.18, Fig. 3a]. However, the plasma IL-7 concentrations in the HIV/Tb co-infected group was higher than that in the mono-HIV infected group following median 16 (12, 22) months of ART [3.28 (2.62–3.62) log_2_ pg/ml vs. 2.21 (1.41–2.90) log_2_ pg/ml, *P *= 0.01, Fig. 3b). Plasma IL-7 concentrations were not associated with the levels of on-ART HIV DNA both at baseline or after ART in the mono-HIV infected group and HIV/Tb co-infected group (Fig. 3c–f).

### Correlations of plasma IDO activities with the levels of on-ART HIV DNA

**Fig. 4:**
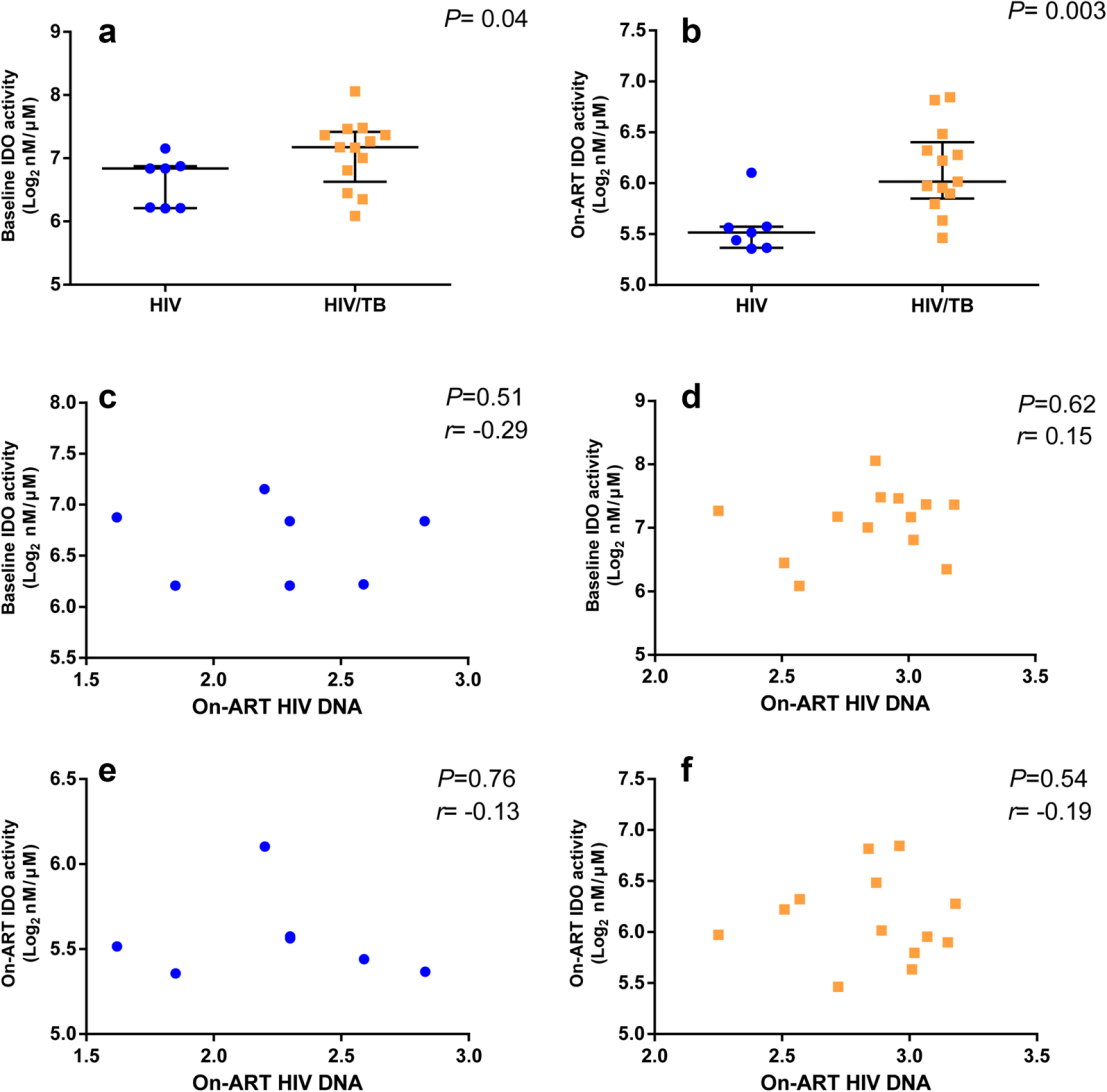
Correlations of plasma IDO activities with the levels of on-ART HIV DNA. The plasma IDO activities in the two groups at baseline (**a**), and after ART (**b**). The correlations between the levels of on-ART HIV DNA and the plasma IDO activities both at baseline and after ART in the mono-HIV infected group and HIV/Tb co-infected group, respectively (**c–f**). All the data analyzed by non-parametric test. The blue dot represents mono-HIV infected patients and the orange dot represents HIV/Tb co-infected patients

The levels of IDO activities while on ART in the PWH co-infected with Tb were significantly difference compared to the mono-HIV infected group at baseline [7.18 (6.63–7.42) log_2_ nM/μM vs. 6.84 (6.21–6.88) log_2_ nM/μM, *P *= 0.04, Fig. 4a]. Besides, after a median 16 (12, 22) months of ART, the plasma IDO activities in the HIV/Tb co-infected group were also higher than in the mono-HIV infected group [6.02 (5.85–6.40) log_2_ nM/μM vs. 5.52 (5.37–5.57) log_2_ nM/μM, *P *= 0.003, Fig. 4b]. Plasma IDO activity was not associated with the levels of on-ART HIV DNA both at baseline or after ART in the mono-HIV infected group and HIV/Tb co-infected group (Fig. 4c–f).

## Discussion

Determining the impact of Tb co-infection on the size of the HIV reservoir may help in understanding how to eradicate HIV in this HIV/Tb co-infected population which accounted for more than 1/3 of the total number of PWH [9]. To the best of our knowledge, our study is the first to demonstrate that Tb co-infection increases the level of total HIV DNA in PBMCs even after Tb is cured, suggesting that Tb maybe related to the increasing of the HIV DNA size in PBMCs. However, studies on this topic are very limited. Interestingly, a recent study from Uganda reported a different result [21] from ours. In that study, levels of cell-associated RNA per HIV DNA template were comparable between the HIV/Tb co-infected persons and mono-HIV infected persons while Tb co-infection significantly decreased the levels of ca-RNA/DNA. The differing results between the Ugandan study and our may be explained by differences in number of patients, which reservoir markers were measured, and the quantification methods employed. However, the most important difference is that baseline HIV viral loads were well matched in their study while they were not in ours. Tb co-infection is known to accelerate HIV replication and is associated with higher HIV viral load in real life [13, 22, 23]. However, viral loads in controls in their study which were comparable to that in HIV/Tb co-infected subjects, suggesting they would be higher than that in the general PWH. Therefore, there might be some other kind of immunodeficiency in their controls so that they failed to suppress HIV replication as the general PWH. The lower levels of ca-RNA in controls compared to the Tb co-infected patients can be explained by this hypothesis. Indeed, previous studies have shown that baseline plasma viral loads are correlated with the size of the HIV reservoir [24–26]. In the current study, pre-ART viral loads, which were also positively correlated with the levels of on-ART HIV DNA, were higher in Tb co-infected persons compared to that in controls. These results may explain why Tb co-infection increased surrogate markers of the size of the HIV reservoir in our study.

Tb co-infection has been shown to contribute to higher levels of systemic immune activation (marked by the increase of multiple pro-inflammatory bio-markers including IL-7) among PWH [11, 23]. In addition, IL-7 has been shown to induce the proliferation of the central memory T cells (TCM) [7, 16, 27] that are the major long-lasting reservoirs in immune responder to ART [28]. Studies have also shown that levels of plasma IL-7 were associated with the speed of HIV reservoir attenuation [7, 16]. In addition, Tb co-infection may enlarge the size of the HIV reservoir by other immunological pathways. Previously, Suchard et al. has reported that plasma IDO activities were suitable as a biomarker of tuberculosis in HIV-positive persons [17]. IDO as an immune checkpoint marker has been widely studied as a target of immunotherapy in the fields of tumor and inflammation biology [29]. Interestingly, we recently observed a positive correlation between IDO activities and the levels of total HIV DNA in PBMCs [18]. In parallel to the levels of HIV DNA, plasma IDO activities were higher both in pre-ART and following ART in a HIV/Tb co-infected population as compared to that in controls. More importantly, IDO activity was shown to be normalized in another study after Tb cured [17]. Based on the above theory, we assumed that Tb could increase the HIV reservoir size through the cytokine-mediated homeostatic proliferation pathway. However, we found there were no correlation between the level of on-ART HIV DNA and plasma IL-7 concentrations, also no correlation with plasma IDO activities. Considering that on-ART HIV DNA in HIV/Tb co-infected group is related to baseline viral load, while on-ART HIV DNA was not related to plasma IL-7 concentrations and IDO activities, we can only found that Tb is associated with increased level of on-ART HIV DNA, which may be related to the higher viral load caused by Tb infection at baseline.

Our results should be interpreted with caution owing to some study limitations. First, the number of participants in both groups was small. Second, surrogate markers of the HIV reservoir were measured by a PCR-based method that may overestimate its size as most of the viral genomes detected are replication-incompetent. Due to the sample size, we did not measure the replication competent HIV by QVOA at the same time, if we do that, the study would be more intact. However, low levels of total HIV DNA are associated with longer-term delays of viral rebound in PWH who have interrupted ART [30–32] indicating its valuable role as an HIV reservoir marker. Interestingly, the drawback of using total HIV DNA as a surrogate marker may also be considered an advantage; defective HIV provirus may also lead to viral antigen production that plays an important role in HIV replication and pathophysiology of this chronic infection [33]. Third, since M.tb only infects macrophages and HIV also can infect macrophages [34], this study mainly evaluated the reservoir size in PBMCs, but lacked data of reservoir size on the difference between CD4 T cells and macrophage. Fourth, because of the limitation of detection methods, latent Tb was difficult to detect, only those who presented with symptoms indicative of Tb were re-tested during the study period in our study. Also, we were not able to distinguish TB from non-TB with the methods available at the study period. Finally, 6 months of TB medication may bias the results of the HIV reservoir size after 1 year’s ART, it may take longer to assess the impact of Tb on the reservoir more appropriately.

## Conclusion

Among the PWH matched for sex, age, baseline CD4 T cell count and baseline CD4/CD8 ratio, those co-infected with Tb had a higher level of viral load at baseline, which was associated with a higher level of HIV DNA after a duration of 16 (12, 22) months’ ART. In other words, Tb is associated with increased surrogate marker of the HIV reservoir. Further studies are warranted to explore the mechanism of Tb co-infection promoting the increasing size of HIV reservoir.

## Data Availability

The datasets used and/or analyzed during the current study are available from the corresponding author on reasonable request.
